# A streamlined pathway for transcatheter aortic valve implantation: the BENCHMARK study

**DOI:** 10.1093/eurheartj/ehae147

**Published:** 2024-03-30

**Authors:** Derk Frank, Eric Durand, Sandra Lauck, Douglas F Muir, Mark Spence, Mariuca Vasa-Nicotera, David Wood, Francesco Saia, Cristóbal A Urbano-Carrillo, Damien Bouchayer, Vlad Anton Iliescu, Christophe Saint Etienne, Florence Leclercq, Vincent Auffret, Lluis Asmarats, Carlo Di Mario, Aurelie Veugeois, Jiri Maly, Andreas Schober, Luis Nombela-Franco, Nikos Werner, Joan Antoni Gómez-Hospital, Julia Mascherbauer, Giuseppe Musumeci, Nicolas Meneveau, Thibaud Meurice, Felix Mahfoud, Federico De Marco, Tim Seidler, Florian Leuschner, Patrick Joly, Jean-Philippe Collet, Ferdinand Vogt, Emilio Di Lorenzo, Elmar Kuhn, Vicente Peral Disdier, Violetta Hachaturyan, Claudia M Lüske, Radka Rakova, Wilbert Wesselink, Jana Kurucova, Peter Bramlage, Gemma McCalmont, for the BENCHMARK Investigator Group, Derk Frank, Gemma McCalmont, Peter Bramlage, Claudia M Lüske, Marie Zielinski, Daniel Greinert, Cornelia Deutsch, Violetta Hachaturyan, Eric Durand, Sandra Lauck, Douglas Muir, Mark Spence, Mariuca Vasa-Nicotera, David Wood, Francesco Saia, Jana Kurucova, Wilbert Wesselink, Radka Rakova, Martin Thoenes, Cristóbal A Urbano-Carrillo, Carlos Lara García, Beatriz Chamorro, Damien Bouchayer, Jean-Philippe Claudel, Hervé Perrier, Viktoria Frebault, Vlad Anton Iliescu, Catalina Andreea Parasca, Christophe Saint Etienne, Katia Lassouani, Florence Leclercq, Wassim Zitouni, Sonia Soltani, Vincent Auffret, Rosalie Le Gal, Lluis Asmarats, Elena Jimenez Xarrie, Carlo Di Mario, Niccolo Ciardetti, Francesco Meucci, Aurelie Veugeois, Imane Bagdadi, Jiri Maly, Lenka Kolinova, Andreas Schober, Georg Delle-Karth, Claudia Schuster, Marie-Christine Leitgeb, Luis Nombela-Franco, Esther Bernardo García, María Aránzazu Ortega Pozzi, Nikos Werner, Jürgen Leick, Michael Lauterbach, Hannah Waschbüsch, Joan Antoni, Guillem Muntané, Julia Mascherbauer Olga Daxböck, Mirela Butkovic, Simona Popescu, Giuseppe Musumeci, Martina Perrero, Nicolas Meneveau, Stephanie Watbled, Thibaud Meurice, Maxence Delomez, Felix Mahfoud, Bruno Scheller, Sebastian Ewen, Ann-Kathrin Berger, Christina Koch, Alexandra Engel, Federico De Marco, Paolo Olivares, Tim Seidler, Frieder Wolf, Carolin Müller, Maren Hünermund, Florian Leuschner, Mathias Konstandin, Lisa Linz, Hannah Ulbricht, Patrick Joly, Sabrina Siame, Jean-Philippe Collet, Nassima Ait Amrane, Ferdinand Vogt, Dow Rosenzweig, Emilio Di Lorenzo, Barbara Cefalo, Elmar Kuhn, Vera Wolf, Vicente Peral Disdier, Jaume Maristany Daunert, Maria Carmen de la Bandera Sanchez

**Affiliations:** Department of Internal Medicine III (Cardiology, Angiology and Intensive Care Medicine), University Clinical Centre Schleswig-Holstein (UKSH), Arnold-Heller Strasse 3, Haus K3, Kiel 24105, Germany; Department of Internal Medicine III (Cardiology, Angiology and Intensive Care Medicine), German Centre for Cardiovascular Research, partner site Hamburg/Kiel/Lübeck, Arnold-Heller Strasse 3, Haus K3, Kiel 24105, Germany; Department of Cardiology, Univ Rouen Normandie, Inserm U1096, CHU Rouen, Rouen, France; Centre for Cardiovascular Innovation, University of British Columbia, Vancouver, BC, Canada; Cardiology Department, James Cook University Hospital, Middlesbrough, UK; Cardiology Department, Mater Private Network, Dublin, Ireland; Cardiology Department, Hospital Sindelfingen-Böblingen, Sindelfingen, Germany; Centre for Cardiovascular Innovation, University of British Columbia, Vancouver, BC, Canada; Department of Cardiology, University of Bologna, Policlinico S. Orsola-Malpighi, Bologna, Italy; Cardiology Department, Hospital Regional Universitario de Málaga, Malaga, Spain; Department of Cardiology, The Clinique de l'Infirmerie Protestante, Lyon, France; Department of Cardiology, University of Medicine and Pharmacy Carol Davila, Bucharest, Romania; Department of Cardiology, Centre Hospitalier Régional Universitaire (CHRU) de Tours, Hôpital Trousseau, Tours, France; Cardiology Department, Montpellier University Hospital, Montpellier University, Montpellier, France; Université de Rennes 1, CHU Rennes Service de Cardiologie, Inserm LTSI U1099, Rennes, France; Servicio de Cardiología, Hospital de la Santa Creu i Sant Pau, Instituto de Investigación Biomédica Sant Pau, Barcelona, Spain; Centro de Investigación Biomédica en Red de Enfermedades Cardiovasculares (CIBERCV), Madrid, Spain; Structural Interventional Cardiology Division, Department of Clinical & Experimental Medicine, Careggi University Hospital, Florence, Italy; Department of Cardiology, Institut Mutualiste Montsouris, Paris, France; Cardiac Center, IKEM Prague, Prague, Czech Republic; Department of Cardiology, Hospital Floridsdorf, Vienna, Austria; Karl Landsteiner Institute for Cardiovascular and Critical Care Research Vienna, Vienna, Austria; Instituto Cardiovascular, Hospital Clínico San Carlos, Instituto de Investigación Sanitaria del Hospital Clínico San Carlos (IdISSC), Madrid, Spain; Medical Department III, Heart Center Trier, Krankenhaus der Barmherzigen Brüder, Trier, Germany; Heart Diseases Institute, Bellvitge University Hospital—IDIBELL, University of Barcelona, Barcelona, Spain; Department of Internal Medicine 3/Cardiology, University Hospital St. Pölten, St. Pölten, Austria; Struttura Complessa of Cardiology, Ospedale Mauriziano, Torino, Italy; Cardiology, Besancon Regional University Hospital Center, Besancon, France; Cardiology, Polyclinique Du Bois, Lille, France; Internal Medicine III, Cardiology, Angiology and Internal Intensive Care Medicine, University Hospital of Saarland, Homburg, Germany; Cardiology, Centro Cardiologico Monzino, Milan, Italy; Department of Cardiology and Pulmonology, Georg-August-University, Göttingen, Germany; Department of Cardiology, University Medicine Göttingen, Heart Center, Göttingen, Germany; Department of Cardiology, Kerckhoff-Klinik, Bad Nauheim, Germany; Department of Medicine III, University of Heidelberg, German Centre for Cardiovascular Research (DZHK), Heilderberg, Germany; Department of Interventional Cardiology, Hôpital Saint Joseph, Marseille, France; Department de Cardiologie, Hôpital de Pitié-Salpêtrière AP-HP, Paris, France; Department for Cardiovascular Surgery, Artemed Klinikum München, München, Germany; Division of Cardiology, Department of Cardiovascular Surgery, L’Ospedale S.Giuseppe Moscati di Avellino, Avellino, Italy; Department of Cardiothoracic Surgery, Heart Center, Faculty of Medicine, University Hospital of Cologne, Cologne, Germany; Cardiology Department, University Hospital Son Espases, Palma de Mallorca, Spain; Institute for Pharmacology and Preventive Medicine, Cloppenburg, Germany; Institute for Pharmacology and Preventive Medicine, Cloppenburg, Germany; Edwards Lifesciences, Prague, Czech Republic; Edwards Lifesciences, Prague, Czech Republic; Edwards Lifesciences, Prague, Czech Republic; Institute for Pharmacology and Preventive Medicine, Cloppenburg, Germany; Cardiology Department, James Cook University Hospital, Middlesbrough, UK; Edwards Lifesciences, Nyon, Switzerland

**Keywords:** Aortic stenosis, Quality of care, Prospective registry, Transcatheter aortic valve implantation, TAVI, Clinical care, Health services

## Abstract

**Background and Aims:**

There is significant potential to streamline the clinical pathway for patients undergoing transcatheter aortic valve implantation (TAVI). The purpose of this study was to evaluate the effect of implementing BENCHMARK best practices on the efficiency and safety of TAVI in 28 sites in 7 European countries.

**Methods:**

This was a study of patients with severe symptomatic aortic stenosis (AS) undergoing TAVI with balloon-expandable valves before and after implementation of BENCHMARK best practices. Principal objectives were to reduce hospital length of stay (LoS) and duration of intensive care stay. Secondary objective was to document patient safety.

**Results:**

Between January 2020 and March 2023, 897 patients were documented prior to and 1491 patients after the implementation of BENCHMARK practices. Patient characteristics were consistent with a known older TAVI population and only minor differences. Mean LoS was reduced from 7.7 ± 7.0 to 5.8 ± 5.6 days (median 6 vs. 4 days; *P* < .001). Duration of intensive care was reduced from 1.8 to 1.3 days (median 1.1 vs. 0.9 days; *P* < .001). Adoption of peri-procedure best practices led to increased use of local anaesthesia (96.1% vs. 84.3%; *P* < .001) and decreased procedure (median 47 vs. 60 min; *P* < .001) and intervention times (85 vs. 95 min; *P* < .001). Thirty-day patient safety did not appear to be compromised with no differences in all-cause mortality (0.6% in both groups combined), stroke/transient ischaemic attack (1.4%), life-threatening bleeding (1.3%), stage 2/3 acute kidney injury (0.7%), and valve-related readmission (1.2%).

**Conclusions:**

Broad implementation of BENCHMARK practices contributes to improving efficiency of TAVI pathway reducing LoS and costs without compromising patient safety.

## Structured Graphical Abstract

**Structured Graphical Abstract:**
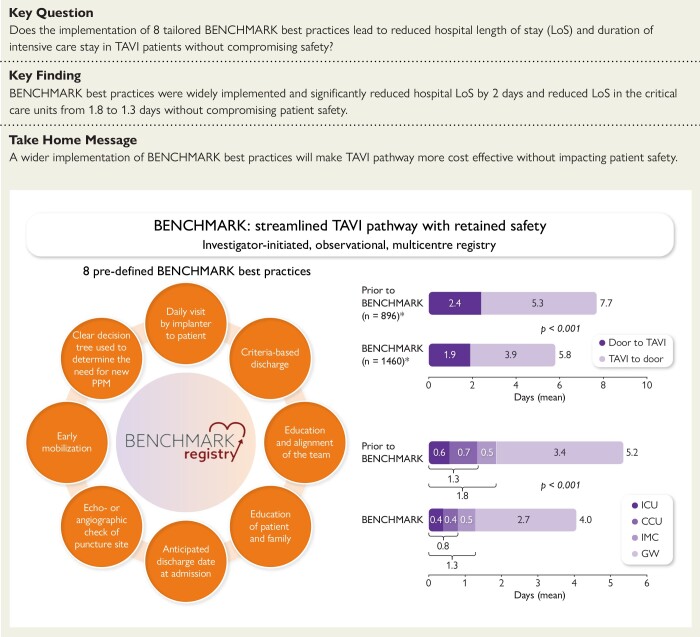
BENCHMARK: streamlined transcatheter aortic valve implantation (TAVI) pathway with retained safety. CCU, coronary care unit; ICU, intensive care unit; GW, general ward; IMC, intermediate care; PPM, permanent pacemaker. *Includes patients with available data on LoS only.

**See the editorial comment for this article ‘Transcatheter aortic valve replacement: benchmarking practices to optimize quality and outcomes', by P. Pibarot, https://doi.org/10.1093/eurheartj/ehae174.**

## Introduction

Transcatheter aortic valve implantation (TAVI) is increasingly used for treating patients with severe symptomatic aortic stenosis (AS) irrespective of risk profile. With its wider adoption, there is an increasing need for streamlined patient pathways to improve efficient patient work-up, treatment, and early discharge without compromising patient safety.

Multiple studies have described factors associated with the potential for early and safe discharge after TAVI, including specific patient characteristics, minimalist peri-procedure approaches, the absence of post-procedural complications such as acute kidney injury (AKI) and conduction disturbances, and early ambulation.^1–9^ The multicentre *Vancouver 3M (multidisciplinary, multimodality, but minimalist)* TAVR study^10^ demonstrated the feasibility and safety of a clinical pathway inclusive of same-day admission for a procedure performed in a cardiac catheterization laboratory or hybrid operating room, local anaesthesia only or with conscious sedation, percutaneous access and closure, removal of a temporary pacemaker at the end of the intervention, standardized post-procedure care with early mobilization, and next-day discharge home.^11^ Study findings confirmed and resulted in excellent safety and efficacy outcomes at 30 days. In addition, cumulative 30-day costs were $11 305/patient lower in the 3M TAVR cohort.^12^ Further, the multicentre *European Feasibility and Safety of Early Discharge After Transfemoral TAVI (FAST-TAVI)* registry demonstrated that pre-specified risk criteria can be effectively used to identify patients in an all-comers collective suitable for safe early discharge and to identify others who may require a longer length of stay (LoS).^13,14^ This led to the concept of a dedicated TAVI coordinator or TAVI nurse, a suggestion that has been widely adopted in North America and other international regions.^11,15^ In a recent assessment (COORDINATE study), the implementation of a TAVI coordinator led to increase in patient satisfaction and improved discharge management without compromising patient safety at 30 days.^16^

This evidence informed the development of the BENCHMARK study to measure the impact of eight tailored BENCHMARK best practices on hospital LoS and duration of critical care and patient satisfaction without compromising patient safety.

## Methods

BENCHMARK (ClinicalTrials.gov Identifier: NCT04579445) is a multicentre, international study of patients with severe symptomatic AS undergoing TAVI at 28 centres across Europe including Austria, France, Germany, Italy, Czech Republic, Romania, and Spain between January 2020 and March 2023 (primary completion of the 30-day follow-up).^17^ Each participating centre was required to establish a physician lead and non-physician TAVI coordinator. At baseline, 13 centres had such a function already established and all centres (*n* = 28) had it established with the implementation of BENCHMARK (named TAVI coordinator in 19 centres, study nurse in 5 centres, research assistant in 1 centre, and other non-physician staff in 3 centres). BENCHMARK was conducted according to the European Medical Device Regulations and International Organization for Standardization (ISO 14155:2020) and the Declaration of Helsinki. BENCHMARK was approved by the independent ethics review boards at each participating site, and all patients provided written informed consent.

### Patients

Consecutive patients with severe symptomatic AS who underwent transfemoral TAVI with a balloon-expandable valve were retrospectively enrolled prior to and prospectively after the introduction of the BENCHMARK best practices. Valve choice was considered to be appropriate for two reasons: (i) Edwards Lifesciences, the maker of a balloon-expandable valve, agreed to provide funding, and the majority of evidence on streamlining of the patient pathway was generated using balloon-expandable valves. In reality, only SAPIEN 3 and SAPIEN 3 Ultra valves were used. (ii) A single valve type was considered to result in less variation with respect to patient outcomes, such as pacemaker rates, which may result in an outcome bias if unevenly distributed. Across all participating centres, the mean proportion of balloon-expandable valves was 59.4%. Patients were required to be 18 years and older and were scheduled for follow-up visits at 30 days and 12 months after the procedure. Patients undergoing valve-in-valve procedures, who were pregnant, or for whom key variables for the assessment were missing (only for patients prior to the implementation) were excluded.

### BENCHMARK best practices

A total of eight best practices were implemented^17^, which included (i) tailored education of patient and family, (ii) determination of an anticipated discharge date at admission based on pre-procedural risk stratification and scheduling of post-procedural diagnostics, (iii) echocardiographic or angiographic check at the end of the procedure to confirm vascular access closure and absence of peri-procedural complications, (iv) nurse-led early mobilization (mobilization of the patient with the help of a nurse done 4–6 h after the intervention in the absence of complications), (v) use of a decision tree to determine the need for new pacemaker implantation if required without increasing hospital stay, (vi) criteria-based discharge, (vii) daily visit by implanting physician and interaction with the rest of the team, and (viii) education and alignment of the internal team (medical, nursing, and paramedical).

Prior to the BENCHMARK Education Phase, each centre identified a defined leadership team (i.e. multidisciplinary heart team) and self-assessed their current alignment of hospital performance with the BENCHMARK best practices. In the Education Phase, centres underwent online education on the BENCHMARK best practices led by their dedicated faculty member. After that, each centre had a post-education call to define the action plan with a designated mentor. During the Implementation Phase consisting of a 2-month time window, each centre introduced the tailored best practices into their hospital routine. Follow-up calls were arranged between the BENCHMARK education team and each centre every 2 weeks to offer assistance with regard to implementation progress. A further follow-up call was held after 30% of the aimed patient number had been included to allow further action plan development in case not all BENCHMARK best practices were not implemented until then.

### Study objectives

The principal objective of BENCHMARK was to document whether the implementation of tailored BENCHMARK best practices into the patient pathway decreases the total hospital LoS and reduces duration of critical care stay [defined as intensive care unit (ICU), cardiac/coronary care unit (CCU), or intermediate care (IMC]). Intensive care unit and CCU were considered units of intensified care when 24 h critical care or life support was required. When the unit was specialized to look after cardiac patients, the unit was classified as a CCU. A patient was considered to be an IMC patient if he/she was not a patient on a general ward nor a patient of the ICU/CCU. Secondary objectives included the assessment of procedural success and complications, patient safety at discharge and 30 days, and patient quality of life (QoL).

### Data collection

Collected data included physical assessments, medical history and symptoms, diagnostic procedures, electrocardiography (ECG), echocardiography (Echo), hospitalization and procedural duration, safety parameters, QoL measures, patient satisfaction surveys, as well as parameters on resource utilization. Frailty was assessed using comprehensive assessment of cognition (The Mini Mental State Exam-2) and activities of daily living (The Katz Activities of Daily Living).^18,19^ The depth of sedation was defined as follows: minimal (anxiolysis), moderate (sleepy and relaxed but can follow simple instructions if asked and may remember parts of the procedure; breathing should not be affected), and deep sedation (sleeping through most of the procedure and unlikely to have recall). Quality of life was assessed using the Toronto Aortic Stenosis Quality of Life Questionnaire (TASQ).^20^ Patients treated with BENCHMARK best practices were interrogated how they assessed different domains of their treatment using a tailored questionnaire. No data on the size of the implanted valve, contrast, and fluoroscopy/radiation dose were collected in an attempt to balance the size of the electronic case report form (eCRF) against the specific purpose of the study. Data were captured in an eCRF (Software for Trials Europe GmbH, Berlin, Germany) and checked for plausibility and completeness.

### Statistical analysis

All available patient data were analysed and missing data were not imputed. Continuous variables were presented as mean ± standard deviation (SD) or median and interquartile ranges (IQR). The Kolmogorov–Smirnov test was used to test for normal distribution. Categorical variables were reported as frequencies and percentages. Comparisons were made using a *t*-test or Mann–Whitney *U* test for continuous variables and Pearson’s χ^2^ or Fisher’s exact test for categorical variables. The interaction tests were performed using univariate analysis of variance for continuous variables and logistic regression for categorical variables. Adjustments for differences in baseline characteristics were performed for the primary outcome using a linear regression model, and the significance of independent variables and their interactions was assessed using analysis of variance (ANOVA) with type III sum of squares. The effect size of continuous variables for adjustment was calculated using Cohen’s *d*. A *P*-value of <.05 was considered statistically significant. All analyses were performed using IBM SPSS Statistics version 29 (IBM, Armonk, NY, USA) or R Core Team (https://www.R-project.org/).

## Results

We included 897 patients treated prior to and 1491 patients treated after the implementation of the BENCHMARK best practices. Patient characteristics were consistent with a known older TAVI population (*Table 1*). The mean age of the total BENCHMARK population was 79.9 ± 6.8 years, 39.4% were female, and 58.5% had New York Heart Association (NYHA) functional class III or IV with a mean EuroSCORE II of 4.8 ± 5.8%. The baseline profile of patients documented prior to and after BENCHMARK implementation was largely comparable. Patients documented prior to BENCHMARK implementation had a slightly higher body mass index (BMI; 27.9 vs. 27.5 kg/m^2^, *P* = .046), higher EuroSCORE II (5.0 vs. 4.7%, *P* < .001), and higher presence of angina CCS 3 or 4 (6.1% vs. 4.1%, *P* = .030) and peripheral artery disease (17.3% vs. 13.8%, *P* = .021). Electrocardiogram and echocardiography-based criteria were mostly similar between groups (*Table 2*). In the total population, atrial fibrillation was present in 18.3%. Pacemaker implantation prior TAVI was present in 9.4% of patients. Pre-existing second-degree atrio-ventricular block was documented in 0.3%; 17.3% had a left ventricular ejection fraction below 50%, and the mean aorto-ventricular pressure gradient was 45.9 mmHg.

**Table 1:** Patient characteristics

	Prior to BENCHMARK		BENCHMARK		
	*N*	Mean ± SD, median (IQR) or *n* (%)	*N*	Mean ± SD, median (IQR) or *n* (%)	*P*-value
Age (years)	897	79.8 ± 6.6 81.0 (76.0; 84.0)	1490	79.9 ± 6.8 81.0 (76.0; 85.0)	.902
Female gender	897	369 (41.1)	1491	571 (38.3)	.152
Body mass index (kg/m^2^)	893	27.9 ± 5.1 27.4 (24.5; 30.7)	1487	27.5 ± 4.9 27.0 (24.2; 30.1)	.046
Aortic valve-related symptoms					
Dizziness with exertion	893	224 (25.1)	1472	360 (24.5)	.732
(Pre-)syncope	893	80 (9.0)	1472	163 (11.1)	.101
NYHA class III or IV	890	543 (61.0)	1473	840 (57.0)	.057
Angina CCS 3 or 4	892	54 (6.1)	1472	60 (4.1)	.030
Risk scores and frailty					
EuroSCORE II	877	5.0 ± 4.8 3.5 (1.8; 5.9)	1448	4.7 ± 6.3 2.8 (1.7; 4.8)	<.001
Frailty, severe	894	37 (4.1)	1481	40 (2.7)	.055
Impaired mobility	890	123 (13.8)	1451	174 (12.0)	.197
Cognitive deficit	894	38 (4.3)	1481	50 (3.4)	.274
Comorbidities					
Previous myocardial infarction	896	131 (14.6)	1479	204 (13.8)	.574
Peripheral artery disease	896	1545 (17.3)	1479	204 (13.8)	.021
Diabetes mellitus	890	284 (31.9)	1451	446 (30.7)	.552
Pulmonary hypertension	808	52 (6.4)	1304	96 (7.4)	.418
Renal insufficiency	896	238 (26.6)	1481	372 (25.1)	.435
Prior permanent pacemaker	895	75 (8.4)	1482	102 (6.9)	.178

SD, standard deviation; IQR, interquartile range; NYHA, New York Heart Association; CCS, Canadian Cardiovascular Society.

**Table 2:** Electrocardiogram and echocardiography at baseline

	Prior to BENCHMARK		BENCHMARK		
	*N*	Mean ± SD or *n* (%)	*N*	Mean ± SD or *n* (%)	*P*-value
**Electrocardiogram (ECG)**					
Rhythm	873		1448		.824
Sinus rhythm		643 (73.7)		1071 (74.0)	
Atrial fibrillation		156 (17.9)		268 (18.5)	
Pacemaker rhythm		63 (7.2)		95 (6.6)	
Other		11 (1.3)		14 (1.0)	
AV block second or third degree	864	12 (1.4)	1445	15 (1.0)	.448
Left bundle branch block	873	110 (12.6)	1450	141 (9.7)	.031
Right bundle branch block	873	97 (11.1)	1450	171 (11.8)	.618
**Echocardiography**					
Left ventricular ejection fraction (LVEF) < 50%	862	157 (18.2)	1462	245 (16.8)	.370
Aortic regurgitation, mod/severe	851	165 (19.4)	1442	274 (19.0)	.820
Aorto-ventricular mean pressure gradient	827	45.5 ± 13.1 44.0 (39.0; 52.0)	1382	46.1 ± 13.8 45.0 (39.0; 53.0)	.658
Aorto-ventricular peak pressure gradient	683	71.7 ± 18.6 71.0 (62.0; 81.0)	1061	73.5 ± 20.6 72.0 (62.0; 84.0)	.244
*V*_max_	644	4.2 ± 0.8 4.2 (4.0; 4.6)	1091	4.2 ± 0.8 4.2 (3.9; 4.6)	.987
Aortic valve area (cm^2^)	714	0.76 ± 0.32 0.73 (0.60; 0.90)	1181	0.77 ± 0.24 0.75 (0.62; 0.90)	.188
Aortic valve area indexed (cm^2^/m^2^)	711	0.41 ± 0.17 0.39 (0.32; 0.46)	1183	0.41 ± 0.14 0.40 (0.33; 0.47)	.276

SD, standard deviation.

### Implementation of BENCHMARK best practices

**Figure 1:**
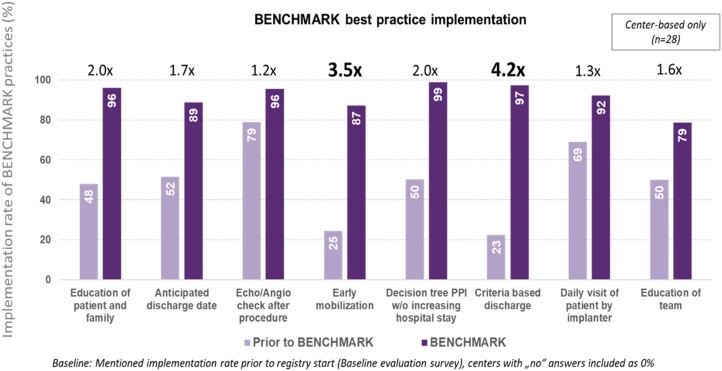
BENCHMARK best practices implementation rate. PPI, permanent pacemaker implantation

The majority of centres had already adopted the echo-/angiographic check (79%), the daily visit of patient by implanter (69%), and anticipated discharge date planning (52%) at baseline (*Figure 1*). With the BENCHMARK practice implementation, ‘criteria-based discharge’ use increased by a factor of 4.2 (97% vs. 23% at baseline), ‘early mobilization’ by a factor of 3.5 (87% vs. 25%), ‘education of patient and family’ (96% vs. 48%) by a factor of 2.0, and the use of a ‘pacemaker decision tree’ (99% vs. 50%) also by a factor of 2.0. BENCHMARK best practices whose use significantly increased in patients with a short LoS (≤median) were tailored education of patient and family, determination of an anticipated discharge date, use of a decision tree to determine the need for new PPM, early mobilization, criteria-based discharge, and daily visit to patient by implanter (see Supplementary data online, *Table S1*).

### Principal objective: length of stay

**Figure 2:**
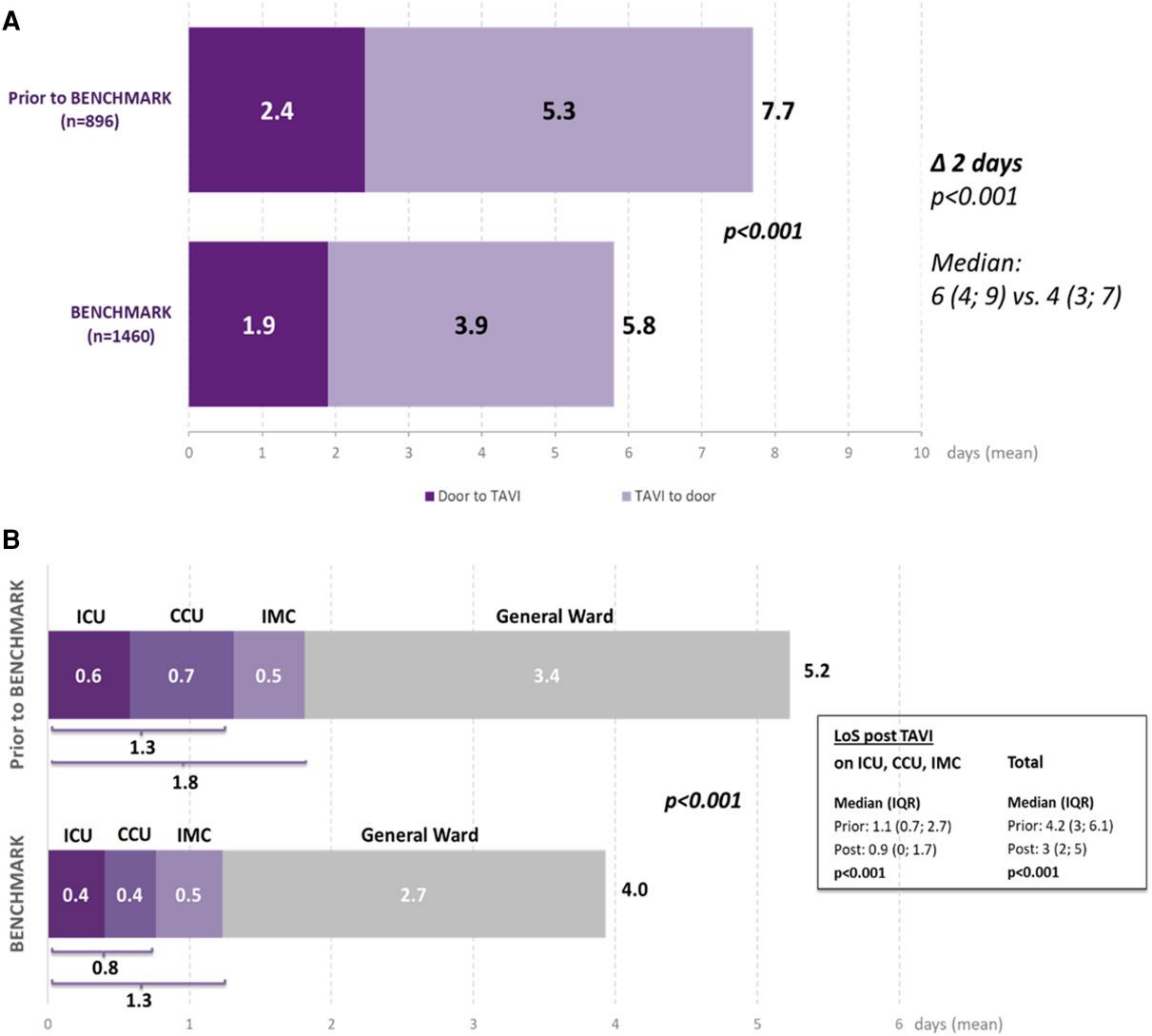
Results for the primary objective: hospital length of stay broken down into door to needle and needle to door (*A*) and length of ICU, CCU, IMC, and general ward stay (*B*). ICU, intensive care unit; CCU, coronary care unit; IMC, intermediate care; GW, general ward

The implementation of BENCHMARK best practices resulted in a reduction in the mean LoS (time from admission to discharge) by ∼2 days from 7.7 ± 7.0 to 5.8 ± 5.6 days (median 6 vs. 4 days; *P* < .001) (*Figure 2A*). Both the time from admission to TAVI (*P* = .040 based on median) and the time from TAVI to discharge (*P* < .001 based on the median) were significantly reduced.

The total LoS across all critical care units decreased from 1.8 to 1.3 days (median 1.1 vs. 0.9 days; *P* < .001). The mean time spent in the ICU was reduced from 0.6 to 0.4 days and the time in the CCU from 0.7 to 0.4 days (*Figure 2B*); 3.4 days prior to and 2.7 days post-BENCHMARK implementation were spent on the general ward. Overall, the mean LoS after the intervention was reduced from 5.2 to 4.0 days (median 4.2 vs. 3.0 days; *P* < .001).

To further explore the effect of the BENCHMARK best practices on total LoS and LoS in ICU/CCU/IMC, we performed the analysis adjusting for baseline variables with a *P*-value of ≤.1 - NYHA class III or IV, angina CCS 3 or 4, severe frailty, and peripheral artery disease; the effect sizes for EuroSCORE II, BMI, and aorto-ventricular peak pressure gradient were small (Cohen’s *d* < 0.2), so these variables were not included. After the adjustment, the reduction in both total LoS (*P* < .001) and LoS in ICU/CCU/IMC (*P* < .001) after the BENCHMARK implementation remained significant. The reduction in LoS (overall and in ICU, CCU, and IMC) was also observed in subgroups defined by patient gender (male/female) and age (>75/≤75 years) (see Supplementary data online, *Table S2*). Similarly, the implementation of BENCHMARK best practices led to the highest reduction in LoS in the subgroup of patients with a lower EuroSCORE.

### Secondary objectives: procedural and 30-day outcomes

Implementation of the BENCHMARK best practices was associated with an increased use of local anaesthesia with or without conscious sedation (96.1 vs. 84.3%; *P* < .001) and reduction in deep sedation (8.2% vs. 20.3%) (*Table 3*). A minimalist anaesthesia strategy was significantly associated with overall LoS (*P* = .031) but had no effect on the LoS in critical care (*P* = .897) (see Supplementary data online, *Table S3*). There were significant decreases in procedure time (vascular access puncture to sheath removal/closure: median 47 vs. 60 min; *P* < .001) and intervention time (start of sedation to patient exit: median 85 vs. 95 min; *P* < .001) following the implementation of change.

**Table 3:** Procedural details and outcomes

	Prior to BENCHMARK		BENCHMARK		
	*N*	Median (IQR) or *n* (%)	*N*	Median (IQR) or *n* (%)	*P*-value
**Local anaesthesia (LA) ± conscious sedation**	896	755 (84.3)	1482	1424 (96.1)	<.001
LA w/o sedation/anxiolytic		57 (7.6)		138 (9.7)	
LA + minimal sedation/anxiolytic		336 (44.6)		616 (43.4)	
LA + moderate sedation		208 (27.6)		550 (38.7)	
Deep sedation		153 (20.3)		116 (8.2)	
**Implanted heart valve**	896		1482		<.001
SAPIEN 3		607 (67.7)		827 (55.8)	
SAPIEN 3 Ultra		289 (32.3)		653 (44.1)	
Other valves		0		2 (0.1)	
**Procedural information**					
Total procedure time	817	60 (40.5; 84)	1391	47 (33; 70)	<.001
Intervention time	815	95 (60; 120)	1402	85 (55; 120)	<.001
**Procedural success**	892	887 (99.4)	1470	1456 (99.0)	.301
Absence of procedural mortality	894	894 (100)	1470	1467 (99.8)	.177
Correct positioning of single valve into proper anatomical location	894	894 (100)	1470	1467 (99.8)	.177
Intended performance of valve	892	888 (99.6)	1470	1459 (99.3)	.374
Device malfunction	894	2 (0.2)	1470	1 (0.1)	.303
**Complications**					
Procedure aborted before device introduced	896	0	1480	1 (0.1)	1.000
Second valve needed	896	5 (0.6)	1481	7 (0.5)	.776
Permanent pacemaker implanted	895	69 (7.7)	1482	87 (5.9)	.079
Conversion to conventional surgery	881	6 (0.7)	1429	4 (0.3)	.195
Bleeding	891	36 (4.0)	1459	31 (2.1)	.007

SD, standard deviation.

Procedural success was >99% with no significant differences between groups. Bleeding rates decreased from 4.0% to 2.1% (*P* = .007).

At discharge and at 30 days, there was no significant difference in safety outcomes between patient groups (*Table 4*). There was a lower rate of major vascular complications in patients after BENCHMARK practice implementation both at discharge (1.8% vs. 3.2%; *P* = .024) and 30 days (2.0% vs. 3.9%; *P* = .007). Similarly, the rates of permanent pacemaker implantation were lower after the implementation of BENCHMARK best practices at discharge (13.2% vs. 17.3%, *P* = .007) and at 30 days (14.0% vs. 19.1%, *P* = .001). Valve-related symptoms or worsening heart failure requiring re-hospitalization after discharge were low with no significant differences between groups (1.1% vs. 1.5%, *P* = .405).

**Table 4:** Outcomes in-hospital and at 30 days (cumulative)

	In-hospital					30-day follow-up				
	Prior to BENCHMARK		BENCHMARK			Prior to BENCHMARK		BENCHMARK		
	*N*	*n* (%)	*N*	*n* (%)	*P*-value	*N*	*n* (%)	*N*	*n* (%)	*P*-value
All-cause mortality	896	2 (0.2)	1460	7 (0.5)	.497	816	4 (0.5)	1230	9 (0.6)	.500
TAVI related		2		4			2		4	
Cardiac, not TAVI related		0		3			0		3	
Non-cardiac		0		0			2		2	
Stroke/TIA	895	7 (0.8)	1453	13 (0.9)	.773	808	10 (1.2)	1311	18 (1.4)	.791
Life-threatening bleeding	894	10 (1.1)	1453	13 (0.9)	.593	809	11 (1.4)	1307	16 (1.2)	.782
AKI (stage 2/3, incl. dialysis)	894	2 (0.2)	1453	9 (0.6)	.173	808	4 (0.5)	1306	10 (0.8)	.456
Coronary artery obstruction requiring intervention	895	3 (0.3)	1453	3 (0.2)	.548	803	5 (0.6)	1305	5 (0.4)	.443
Major vascular complication	895	29 (3.2)	1453	26 (1.8)	.024	812	32 (3.9)	1308	26 (2.0)	.007
Permanent pacemaker	897	155 (17.3)	1473	195 (13.2)	.007	897	171 (19.1)	1473	206 (14.0)	.001
Re-hospitalization^a^										
Valve-related symptoms or worsening CHF						809	12 (1.5)	1305	14 (1.1)	.405
Other reasons						809	44 (5.4)	1305	55 (4.2)	.195

TAVI, transcatheter aortic valve implantation; TIA, transient ischaemic attack; AKI, acute kidney injury; CHF, congestive heart failure.

^a^Not applicable at discharge.

The rates of all-cause mortality and valve-related hospitalization were not significantly different in subgroups defined by sex (male/female), age (>75/≤75 years), and EuroSCORE II (low, intermediate, and high) (see Supplementary data online, *Table S4*).

### Secondary objectives: patients’ quality of life and satisfaction

**Figure 3:**
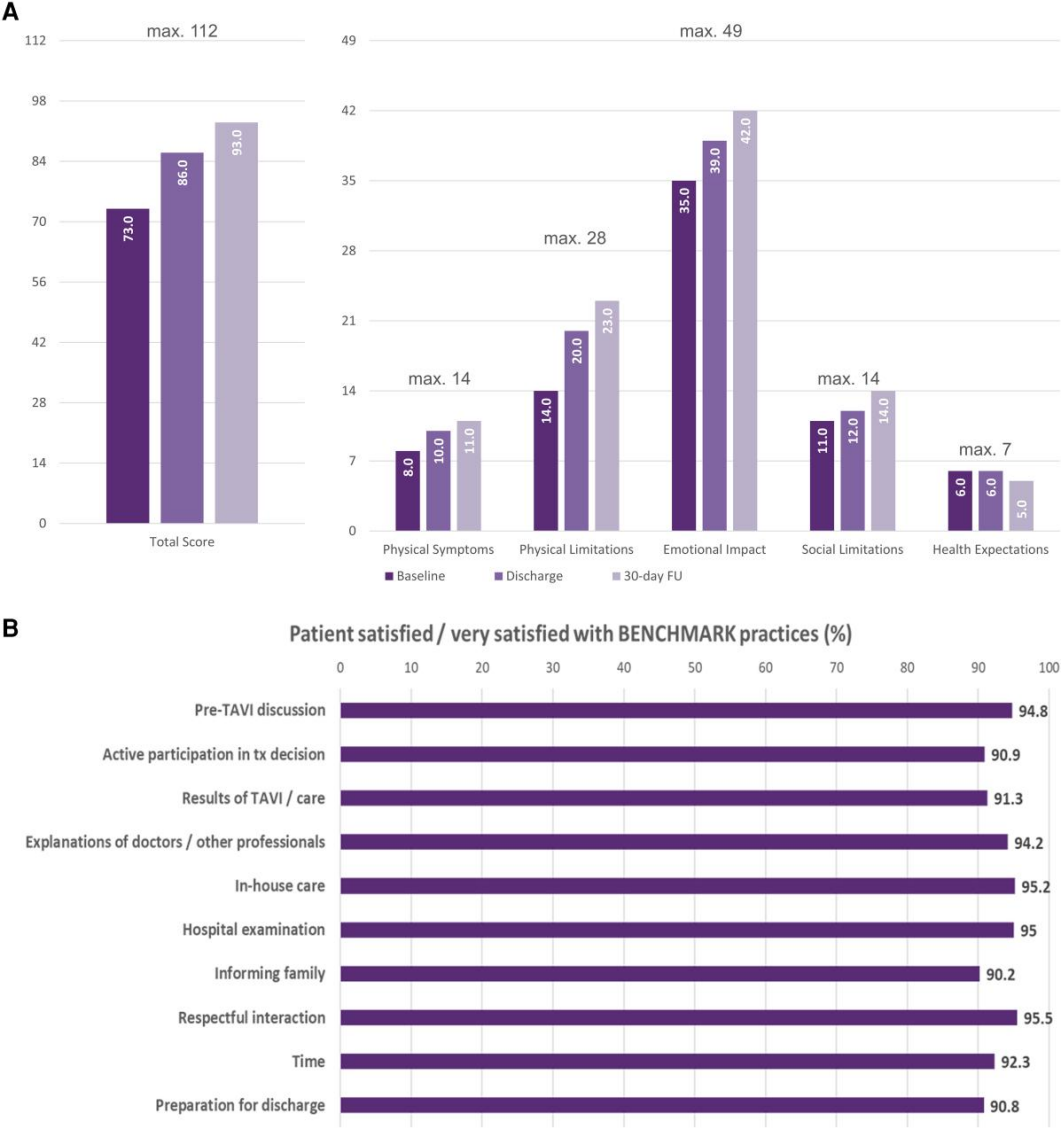
Improvement in quality of life based on TASQ (*A*) and patient satisfaction with the BENCHMARK approach (*B*). TASQ, Toronto Aortic Stenosis Questionnaire; FU, follow-up

Quality of life was measured after the implementation of BENCHMARK practices, but not before. Patients reported an improvement of their QoL based on the TASQ questionnaire from hospital admission (median 73 points) to discharge (median 86 points) and at 30-day follow-up (median 93 points; *P* < .001) (*Figure 3A*). The same pattern was observed on patients’ self-reported physical symptoms, physical limitations, emotional impact, and social limitations subscales. Health expectations were unaffected.

Patients reported they were overall either satisfied or very satisfied with their treatment in more than 90% of all domains with the items ‘respectful interaction’ (95.5%), ‘in-house care’ (95.2%), ‘hospital examinations’ (95.0%), and ‘explanation of doctors/other professionals’ (94.2%) reaching the highest agreement (*Figure 3B*).

## Discussion

The results of the BENCHMARK study demonstrate that the incorporation of BENCHMARK best practices led to a significant decrease in LoS by > 25% (or 2 days) and reduced use of critical care resources without compromising patient safety at hospital discharge and at 30-day follow-up (*Structured Graphical Abstract*).

### Hospital length of stay and use of critical care resources

The reduction of hospital LoS has both clinical and economic benefits. In the present study, we demonstrated a significant decrease in the total hospital LoS (admission to discharge) by 2 days after the implementation of the BENCHMARK best practices. Furthermore, the LoS from admission to TAVI, and TAVI to discharge, including ICU and CCU stay, was also significantly reduced.

The length of hospital stay in BENCHMARK in general was also reported to be shorter compared with the results of study from the West German Heart and Vascular Centre.^21^ Although authors reported a reduction in the total length of stay after the implementation of a TAVI coordinator, the time from admission to implantation was 6 days compared with 1.9 days in our study, while the time from TAVI to discharge was 7 days, compared with 5.8 days reported in BENCHMARK. The Vancouver 3M TAVR study^10^ reported that, based on the use of objective screening criteria as well as streamlined peri- and post-procedural management, the proportion of patients being safely discharged home the day after the intervention reached 80.1%, which is higher than in the BENCHMARK data set. It is important to note, however, that BENCHMARK is an all-comer data set, whereas in the Vancouver 3M TAVR study, the number of enrolled patients (*n* = 411) was much lower than the number of screened patients (*n* = 1400). The French FAST TAVI II study,^22^ which studied patients discharged within 3 days after the intervention as the primary endpoint, reported an increase in the rate of patients being discharged early (58.1 vs. 42.3%; *P* < .0001) after introducing 10 distinct quality of care measures targeting logistic domains and the prevention of complications. The numbers compare well with the shortening of the post-procedural stay from 5.3 to 4.0 days (median 4 vs. 3 days; *P* < .001) in the BENCHMARK study. Efficient discharge planning plays a critical role in identifying suitable candidates for a safe early discharge after TAVI. One of the BENCHMARK practices included an implementation of an early discharge decision protocol, which was adapted from the FAST-TAVI registry.^14^ Pre-specified criteria were considered after TAVI to define a low-risk population for early discharge, including factors such as NYHA class ≤ II, patients having complications on Day 0 to 1 but free of signs or symptoms on Day 3, independent mobilization and self-caring, as well as the absence of unresolved AKI type 3, paravalvular leakage, stroke/transient ischaemic attack (TIA), and haemodynamic instability. The implementation of this protocol has shown that the majority of unselected patients undergoing TAVI can be safely discharged early after the intervention and approximately one-third of patients were discharged later due to logistical and not medical reasons.^13^

Due to the fact that a substantial number of patients in both phases in our registry was recruited during the COVID-19 pandemic period (pre-BENCHMARK, 2020–22; BENCHMARK, 2021–23), there was a certain impact of the pandemic which needs to be acknowledged. However, although one may expect a prolonged length of hospital stay, several studies have shown that the LoS in TAVI patients was either comparable or shorter during the COVID-19 pandemic compared with the pre-pandemic period.^23–27^ Thus, we may only speculate that the pandemic itself had an effect on the TAVI LoS and outcomes in patients in either phase.

### Procedural and 30-day outcomes

Technical success at the exit from the procedure room was reported for 99% of the patients irrespective of the implementation of the BENCHMARK practices. The relevant reduction of procedural time is noteworthy and potentially associated with a reduction of contrast amount and fluoroscopy/radiation dose, although no specific data were collected. Adverse outcomes at discharge and 30 days were low with a 30-day mortality of only 0.6% in the BENCHMARK group vs. 0.5% prior to its implementation. Furthermore, rates of stroke/TIA, life-threatening bleeding, stage II/III AKI, and coronary artery obstruction were all low. Importantly, no increase in the rate of hospital readmission was observed after BENCHMARK implementation, despite earlier discharge.

Similarly low complication rates at 30 days were reported in previous studies.^16,21^ The Vancouver 3M TAVR study results also demonstrated that a streamlined patient pathway allowed for safe expedited discharge.^10^ A composite primary endpoint of all-cause mortality or stroke by 30 days occurred in 2.9% of the patients (vs. 2.0% in our data set). Major vascular complications were reported by 2.4% (vs. 2.0% BENCHMARK), readmissions by 9.2% (vs. 5.3% BENCHMARK), and cardiac readmissions by 5.7% (vs. 1.1% BENCHMARK). The results are also in line with previous reports from the FAST-TAVI study, which investigated the effects of close monitoring, early mobilization, accelerated reconditioning, and discharge planning on the appropriacy of early discharge. The study showed that patients appropriately discharged early had very low rates of all-cause mortality, vascular complications (0.3%), permanent pacemaker implantation (4.3%), stroke (0.0%), and major bleeding at 30 days (0.3%).^13,14^ The French FAST TAVI II study^22^ reported a 30-day mortality of 1.0% for the control and 0.5% for the intervention arms, which was not statistically different (*P* = .29) and in a similar order than the mortality in BENCHMARK. Especially the early mobilization of patients after the intervention could be implemented more decisively as rates were on the lower end in BENCHMARK even after the introduction of the best practices.

BENCHMARK was associated with a reduction of clinically relevant bleeding, and the peri-procedural rate was 4.0% prior to BENCHMARK and 2.1% with the implementation of BENCHMARK (*P* = .007). Procedural factors and antithrombotic management may also contribute to individual bleeding susceptibility. There is a six-item score (PREDICT-TAVR) which includes blood haemoglobin, serum iron, creatinine clearance, common femoral artery diameter, and dual antiplatelet as well as anticoagulant therapy.^28^ Unfortunately, none of these variables were captured in BENCHMARK.

Major vascular complications were noted in 3.2% and 1.8% of patients up until discharge (*P* = .024) and 3.9% vs. 2.0% at 30 days (*P* = .007). Major vascular complications were defined as (i) aortic dissection or aortic rupture; (ii) vascular (arterial or venous) injury (perforation, rupture, dissection, stenosis, ischaemia, arterial or venous thrombosis including pulmonary embolism, arteriovenous fistula, pseudoaneurysm, haematoma, retroperitoneal haematoma, infection) or compartment syndrome resulting in death, VARC type ≥ 2 bleeding, limb or visceral ischaemia, or irreversible neurologic impairment; (iii) distal embolization (non-cerebral) from a vascular source resulting in death, amputation, limb or visceral ischaemia, or irreversible end-organ damage; (iv) unplanned endovascular or surgical intervention resulting in death, VARC type ≥ 2 bleeding, limb or visceral ischaemia, or irreversible neurologic impairment; and (v) closure device failure resulting in death, VARC type ≥ 2 bleeding, limb or visceral ischaemia, or irreversible neurologic impairment.^29^ As such, while vascular access bleeding was already prevented by high implementation rates of angiographic check of closure, there are more potential sources which may have led to a reduction of major vascular complications.

Our study provides importance evidence that the eight BENCHMARK best practices can be implemented successfully in diverse clinical contexts without compromising the safety of patients.

### Patients’ quality of life and satisfaction

Improved QoL is one of the major treatment goals in patients with severe aortic stenosis. With the implementation of BENCHMARK practices, patients reported improved QoL at 30 days compared with baseline, with early improvements seen already at discharge. These findings are in line with previous studies that demonstrated substantial early QoL improvements after TAVI.^30–33^ Despite the fact that patients in our study were old and the majority presented with severe symptoms, large improvement in health status and QoL in most patients was still observed after the intervention. Unfortunately, we had no opportunity to collect QoL data prior to the BENCHMARK programme implementation.

BENCHMARK was associated with high patient satisfaction with most patients reporting feeling satisfied or very satisfied with their patient journey. One of the highest satisfaction domains included the explanations regarding the treatment and care and a respectful treatment from the health professionals. This reflects the fact that patients were well informed and prepared for the procedure and post-procedure care, improving their self-reliance, self-efficacy, and active involvement in health-related decisions. Similar findings were reported from the COORDINATE study with high patient satisfaction with the treatment pathway after the introduction of a TAVI coordinator.^16^ Therefore, TAVI coordinators play an essential role in patient education as well as enhanced communication between the heart team and patient and their family.

### Perspectives

The BENCHMARK programme is a multidisciplinary quality improvement project facilitated by peer-to-peer mentorship and the translation of contemporary evidence. The way it is designed today improves the patient pathway in the hospital and what the hospital and its staff may be able to do to streamline the patient pathway and make it more effective while retaining the safety of TAVI. It appears reasonable to enter into a discussion how the BENCHMARK pathway may be further improved.

A potential further objective of such programmes is to further reduce the hospital LoS. Same-day admission and discharge are potentially feasible if patients experience no complications and do not have an increased risk of pacemaker requirements.^34^ While it appears possible in some European countries, there is a reimbursement barrier in others. For example, in Germany, there is a reimbursement for TAVI that assumes a minimum LoS after the procedure. Compared with other countries in the BENCHMARK data set and beyond (USA, Canada, Australia), the hospital stay in Germany is long, but if patients are discharged early in Germany, the hospital may partially loose its full reimbursement. Therefore, from an economic perspective of the hospital, early discharge is of limited value. These hurdles need to be overcome to result in a truly efficient patient pathway for TAVI. Although the economic benefits of shortened inpatient stays and reduced need for intensive bed capacity have not been directly assessed in our study, such analysis is currently being planned.

Finally, going beyond the hospitalization for the procedure, the definition of an AS pathway may be potentially expanded.^35^ The time from diagnosis to referral and from referral to the actual intervention is still very long in some European countries and can certainly be improved in all of them.

### Limitations

While the multinational nature of this study increases the applicability of the findings, the existing inter- and intra-country differences in patient management and treatment need to be considered. Due to the lack of randomization, there is a potential for confounding and bias in the analysis with limited ability for adjustment. Although patients were compared before and after the implementation of the BENCHMARK best practices, the prior patient data were collected retrospectively, which may have introduced bias due to missing data. In addition, QoL, patient satisfaction, and staff working hours were not documented prior to the implementation of the BENCHMARK practices, limiting the assessment of improvements. Lastly, the time window of the study was quite short, making it unlikely that relevant changes in centre proficiency occurred while the study was ongoing. However, we cannot rule out that the study results could be partly attributable to other factors, independently of implementation of the BENCHMARK practices.

## Conclusion

Our findings suggest that the introduction of the BENCHMARK best practices led to a streamlined TAVI patient pathway and reduced procedure time and length of hospital stay, while the overall patient safety at 30 days did not appear to be compromised. As TAVI expands to all severe AS patients, regardless of surgical risk, a wider implementation of the BENCHMARK practices appears warranted and would reduce procedure-related and resource utilization costs as well as other risks associated with prolonged hospitalization.

## Supplementary Material

ehae147_Supplementary_Data

## Data Availability

Available from the corresponding author upon reasonable request.

## Pre-registered Clinical Trial Number

ClinicalTrials Identifier: NCT04579445.

## Appendix

**BENCHMARK Investigator Group**

**Principal Investigators**

Derk Frank (Kiel, Germany)

Gemma McCalmont (Middlesbrough, UK)

**Sponsor Representative and Project Management**

Peter Bramlage (Cloppenburg, Germany)

Claudia M. Lüske (Cloppenburg, Germany)

Marie Zielinski (Cloppenburg, Germany)

Daniel Greinert (Cloppenburg, Germany)

Cornelia Deutsch (Cloppenburg, Germany)

Violetta Hachaturyan (Cloppenburg, Germany)

**Steering Committee**

Eric Durand (Rouen, France)

Sandra Lauck (Vancouver, BC, Canada)

Douglas Muir (Middlesbrough, UK)

Mark Spence (Dublin, Ireland)

Mariuca Vasa-Nicotera (Sindelfingen, Germany)

David Wood (Vancouver, BC, Canada)

Francesco Saia (Bologna, Italy)

**Funder Representatives**

Jana Kurucova (Prague, Czech Republic)

Wilbert Wesselink (Prague, Czech Republic)

Radka Rakova (Prague, Czech Republic)

Martin Thoenes (Geneva, Switzerland)

**Investigators/Centres**

Cristóbal A. Urbano-Carrillo, Carlos Lara García, Beatriz Chamorro (Malaga, Spain)

Damien Bouchayer, Jean-Philippe Claudel, Hervé Perrier, Viktoria Frebault (Lyon, France)

Vlad Anton Iliescu, Catalina Andreea Parasca (Bucharest, Romania)

Christophe Saint Etienne, Katia Lassouani (Tours, France)

Florence Leclercq, Wassim Zitouni, Sonia Soltani (Montpellier, France)

Vincent Auffret, Rosalie Le Gal (Rennes, France)

Lluis Asmarats, Elena Jimenez Xarrie (Barcelona, Spain)

Carlo Di Mario, Niccolo Ciardetti, Francesco Meucci (Florence, Italy)

Aurelie Veugeois, Imane Bagdadi (Paris, France)

Jiri Maly, Lenka Kolinova (Prague, Czech Republic)

Andreas Schober, Georg Delle-Karth, Claudia Schuster, Marie-Christine Leitgeb (Vienna, Austria)

Luis Nombela-Franco, Esther Bernardo García, María Aránzazu Ortega Pozzi (Madrid, Spain)

Nikos Werner, Jürgen Leick, Michael Lauterbach, Hannah Waschbüsch (Trier, Germany)

Joan Antoni Gómez-Hospital, Guillem Muntané (Barcelona, Spain)

Julia Mascherbauer Olga Daxböck, Mirela Butkovic, Simona Popescu (St. Pölten, Austria)

Giuseppe Musumeci, Martina Perrero (Turin, Italy)

Nicolas Meneveau, Stephanie Watbled (Besancon, France)

Thibaud Meurice, Maxence Delomez (Lille, France)

Felix Mahfoud, Bruno Scheller, Sebastian Ewen, Ann-Kathrin Berger, Christina Koch, Alexandra Engel (Homburg, Germany)

Federico De Marco, Paolo Olivares (Milan, Italy)

Tim Seidler, Frieder Wolf, Carolin Müller, Maren Hünermund (Göttingen, Germany)

Florian Leuschner, Mathias Konstandin, Lisa Linz, Hannah Ulbricht (Heidelberg, Germany)

Patrick Joly, Sabrina Siame (Marseille, France)

Jean-Philippe Collet, Nassima Ait Amrane (Pitie Salpetriere, Paris, France)

Ferdinand Vogt, Dow Rosenzweig (Munich, Germany)

Emilio Di Lorenzo, Barbara Cefalo (Avellino, Italy)

Elmar Kuhn, Vera Wolf (Cologne, Germany)

Vicente Peral Disdier, Jaume Maristany Daunert, Maria Carmen de la Bandera Sanchez (Mallorca, Spain)
