# Supplementary material for: A streamlined pathway for transcatheter aortic valve implantation: the BENCHMARK study
Source: Eur Heart J. 2024 Mar 30;45(21):1904–16. doi: 10.1093/eurheartj/ehae147 (PMC11143387; doi:10.1093/eurheartj/ehae147)
Supplement: ehae147_Supplementary_Data [file ehae147_supplementary_data.zip › Sample Size Calculation.docx]

**Sample Size Calculation**

A total number of 2400 TF TAVI patients were estimated to be included into the BENCHMARK registry of which 900 patients were planned to be documented prior to the implementation of BENCHMARK best practices and further 1500 patients were planned to be documented after the implementation.

The sample size estimate was based on the ability to discriminate any changes in outcomes between the retrospective baseline evaluation and prospective effect documentation phase.

| **30 Day** |  | **Retrospective Phase**  **(N = 900)** | **Prospective Phase**  **(N = 1500)** |
| --- | --- | --- | --- |
|  | Assumption | 95%CI | 95%CI |
| Mortality | 1% | ± 0.65 | ± 0.50 |
| Stroke | 1% | ± 0.65 | ± 0.50 |
| Major vascular complications | 1.5% | ± 0.79 | ± 0.62 |
| PPM | 6% | ± 1.55 | ± 1.20 |
| Timely discharge | 80% | ± 2.61 | ± 2.02 |
| Readmission | 4% | ± 1.28 | ± 0.99 |
| **12 Months** |  | **Retrospective Phase**  **(N = 800)*** | **Prospective Phase**  **(N = 1400)*** |
|  | Assumption | 95%CI | 95%CI |
| Mortality | 11.8% | ± 2.24 | ± 1.69 |
| Stroke | 8.0% | ± 1.88 | ± 1.42 |
| Major vascular complications | 8.5% | ± 1.93 | ± 1.46 |
| PPM | 10% | ± 2.08 | ± 1.57 |
| Readmission | 14.8% | ± 2.46 | ± 1.86 |
| * *Due to expected loss during follow-up with not all patients returning to the hospital, this will result in about 800 and 1400 TF TAVI patients respectively for the 12-month visit.*  PPM, Permanent pacemaker implantation | | | |
