# Supplementary material for: A streamlined pathway for transcatheter aortic valve implantation: the BENCHMARK study
Source: Eur Heart J. 2024 Mar 30;45(21):1904–16. doi: 10.1093/eurheartj/ehae147 (PMC11143387; doi:10.1093/eurheartj/ehae147)
Supplement: ehae147_Supplementary_Data [file ehae147_supplementary_data.zip › Supplementary Table 1.docx]

**Supplementary Table 1:** LoS by BENCHMARK best practice implementation

|  | LoS ≤ median | LoS > median | p-value |
| --- | --- | --- | --- |
| Age, years | 79.4 ± 6.8 | 80.2 ± 6.9 | 0.023 |
| EuroScore, % | 3.6 ± 4.7 | 5.7 ± 7.3 | <0.001 |
| Frailty, % | 16 (2.2) | 22 (3.1) | 0.291 |
| Local anesthesia, % | 714 (96.6) | 689 (95.7%) | 0.359 |
| Education of patient and family (1) | 719 (97.3) | 684 (94.9) | 0.017 |
| Determination of an anticipated discharge date (2) | 679 (92.1) | 614 (85.4) | <0.001 |
| Decision tree to determine the need for new PPM (3) | 727 (99.0) | 702 (98.9) | 0.746 |
| Echo- or angiographic check (4) | 685 (92.7) | 710 (98.5) | <0.001 |
| Early mobilization (5) | 676 (91.5) | 598 (82.9) | <0.001 |
| Criteria based discharge (6) | 730 (99.5) | 677 (95.4) | <0.001 |
| Daily visit to patient by implanter (7) | 697 (95.0) | 637 (89.7) | <0.001 |

*Legend:* 1) Education of patient and family; 2) Determination of an anticipated discharge date at admission based on pre‐procedural risk stratification and scheduling of post‐procedural diagnostics; 3) Echocardiographic‐ or angiographic check at the end of procedure immediately performed to confirm vascular access closure and absence of peri-procedural complications; 4) Early mobilization of the patient; 5) Use of a decision tree to determine the need for new pacemaker implantation if required without increasing hospital stay; 6) Criteria-based discharge; 7) Daily visit to patient by implanting physician and interaction with rest of the team

The 8^th^ best practice was implemented on center-level only (not patient-level): 8) Education and alignment of the internal team (medical, nursing and paramedical).
