# Supplementary material for: A streamlined pathway for transcatheter aortic valve implantation: the BENCHMARK study
Source: Eur Heart J. 2024 Mar 30;45(21):1904–16. doi: 10.1093/eurheartj/ehae147 (PMC11143387; doi:10.1093/eurheartj/ehae147)
Supplement: ehae147_Supplementary_Data [file ehae147_supplementary_data.zip › Supplementary Table 2.docx]

**Supplementary Table 2:** Primary endpoint analysis in subgroup gender, EuroSCORE II and patient age

|  | Prior to BENCHMARK | With BENCHMARK implementation | p-value | p-value for interaction |
| --- | --- | --- | --- | --- |
| LoS overall |  |  |  |  |
| Men | 6.0 (4.0, 8.0) | 4.0 (3.0, 7.0) | <0.001 | 0.379 |
| Women | 6.0 (4.0, 9.0) | 4.0 (3.0, 7.0) | <0.001 |  |
| EuroSCORE II low | 6.0 (4.0, 8.0) | 4.0 (3.0, 6.0) | <0.001 | 0.060 |
| EuroSCORE II intermediate | 6.0 (4.0, 9.0) | 6.0 (4.0, 9.0) | 0.103 |  |
| EuroSCORE II high | 6.5 (4.0, 13.0) | 6.0 (4.0, 9.5) | 0.269 |  |
| Patient age ≤75 years | 5 (4.0, 7.0) | 4.0 (3.0, 6.0) | <0.001 | 0.516 |
| Patient age >75 years | 6.0 (4.0, 9.0) | 5.0 (3.0, 7.0) | <0.001 |  |
| LoS (ICU, CCU, IMC) |  |  |  |  |
| Men | 1.1 (0.7, 3.0) | 0.9 (0, 1.4) | <0.001 | 0.700 |
| Women | 1.0 (0.8, 2.1) | 0.9 (0, 1.8) | <0.001 |  |
| EuroSCORE II low | 1.1 (0.8, 2.8) | 0.9 (0, 1.7) | <0.001 | 0.895 |
| EuroSCORE II intermediate | 1.0 (0.4, 2.4) | 0.9 (0.3, 1.3) | 0.011 |  |
| EuroSCORE II high | 1.0 (0, 2.8) | 0.9 (0, 1.8) | 0.152 |  |
| Patient age ≤75 years | 1.1 (0.1, 2.8) | 0.9 (0.1, 1.1) | <0.001 | 0.438 |
| Patient age >75 years | 1.1 (0.8, 2.7) | 0.9 (0, 1.8) | <0.001 |  |

*Legend:* LoS, length of stay
