# Supplementary material for: A streamlined pathway for transcatheter aortic valve implantation: the BENCHMARK study
Source: Eur Heart J. 2024 Mar 30;45(21):1904–16. doi: 10.1093/eurheartj/ehae147 (PMC11143387; doi:10.1093/eurheartj/ehae147)
Supplement: ehae147_Supplementary_Data [file ehae147_supplementary_data.zip › Supplementary Table 3.docx]

**Supplementary Table 3:** Effect of full anesthesia vs. conscious sedation on the LoS

|  | Full anesthesia | Conscious sedation | p-value |
| --- | --- | --- | --- |
| Total population |  |  |  |
| LoS (TAVI to Discharge), days | 4.0 (2.0, 6.0) | 3.0 (3.0, 6.0) | 0.031 |
| LoS intensified care, days | 1.0 (0.4, 1.2) | 1.0 (0, 2.0) | 0.897 |
| Prospective |  |  |  |
| LoS (TAVI to Discharge), days | 4.0 (2.0, 6.0) | 3.0 (2.0, 5.0) | 0.020 |
| LoS intensified care, days | 1.0 (0.2, 1.8) | 0.9 (0, 1.7) | 0.585 |
| Retrospective |  |  |  |
| LoS (TAVI to Discharge), days | 4.0 (3.0, 6.0) | 4.0 (3.0, 6.0) | 0.036 |
| LoS intensified care, days | 1.0 (0.8, 1.1) | 1.1 (0.7, 3.0) | 0.002 |

*Legend:* LoS, length of stay; TAVI, transcatheter aortic valve implantation
