# Supplementary material for: A streamlined pathway for transcatheter aortic valve implantation: the BENCHMARK study
Source: Eur Heart J. 2024 Mar 30;45(21):1904–16. doi: 10.1093/eurheartj/ehae147 (PMC11143387; doi:10.1093/eurheartj/ehae147)
Supplement: ehae147_Supplementary_Data [file ehae147_supplementary_data.zip › Supplementary Table 4.docx]

**Supplementary Table 4:** Patient safety by subgroup

|  | Prior to BENCHMARK | With BENCHMARK implementation | p-value | p-value for interaction |
| --- | --- | --- | --- | --- |
| All-cause mortality |  |  |  |  |
| Men | 0 (0) | 4 (0.5) | 0.123 | 0.554 |
| Women | 2 (0.6) | 3 (0.6) | 0.696 |  |
| EuroSCORE II low | 1 (0.2) | 5 (0.5) | 0.262 | 0.600 |
| EuroSCORE II intermediate | 1 (0.6) | 1 (0.6) | 0.633 |  |
| EuroSCORE II high | 0 (0) | 1 (1.1) | 0.311 |  |
| Patient age ≤75 years | 0 (0) | 1 (0.3) | 0.579 | 0.203 |
| Patient age >75 years | 2 (0.3) | 6 (0.6) | 0.245 |  |
| Re-hospitalisation (valve-related) |  |  |  |  |
| Men | 8 (1.7) | 7 (0.9) | 0.182 | 0.469 |
| Women | 4 (1.2) | 7 (1.5) | 0.751 |  |
| EuroSCORE II low | 7 (1.3) | 9 (0.9) | 0.486 | 0.409 |
| EuroSCORE II intermediate | 5 (3.3) | 4 (2.4) | 0.616 |  |
| EuroSCORE II high | 0 (0) | 1 (1.1) | 0.321 |  |
| Patient age ≤75 years | 2 (1.1) | 2 (0.6) | 0.518 | 0.935 |
| Patient age >75 years | 10 (1.6) | 12 (1.3) | 0.577 |  |

*Legend:* None
